# Traffic-Related Atmospheric Pollutants Levels during Pregnancy and Offspring’s Term Birth Weight: A Study Relying on a Land-Use Regression Exposure Model

**DOI:** 10.1289/ehp.10047

**Published:** 2007-06-01

**Authors:** Rémy Slama, Verena Morgenstern, Josef Cyrys, Anne Zutavern, Olf Herbarth, Heinz-Erich Wichmann, Joachim Heinrich, the LISA Study Group

**Affiliations:** 1 GSF–National Research Center for Environment and Health, Institute of Epidemiology, Neuherberg, Germany; 2 INSERM, Institut National de la Santé et de la Recherche Médicale, UMR822, Le Kremlin-Bicêtre, France; 3 INED, Institut National des Etudes Démographiques, Paris, France; 4 Univ Paris-Sud, Le Kremlin-Bicêtre, France; 5 WZU–Environmental Science Center, University Augsburg, Augsburg, Germany; 6 Kinderklinik und Kinderpoliklinik in Dr. v. Hauner’schen Kinderspital, Munich, Germany; 7 UFZ–Umwelt Forschungszentrum, Human Exposure Research and Epidemiology, Leipzig, Germany; 8 University of Leipzig, Medical Faculty, Leipzig, Germany; 9 Ludwig-Maximilians University of Munich, Institute of Medical Data Management, Biometrics and Epidemiology, Munich, Germany

**Keywords:** atmospheric pollution, birth weight, diesel soot, environment, geographic information system, intrauterine growth restriction, particulate matter, pregnancy, reproduction, road traffic, sensitivity analysis

## Abstract

**Background:**

Some studies have suggested that particulate matter (PM) levels during pregnancy may be associated with birth weight. Road traffic is a major source of fine PM (PM with aero-dynamic diameter < 2.5 μm; PM_2.5_).

**Objective:**

We determined to characterize the influence of maternal exposure to atmospheric pollutants due to road traffic and urban activities on offspring term birth weight.

**Methods:**

Women from a birth cohort [the LISA (Influences of Lifestyle Related Factors on the Human Immune System and Development of Allergies in Children) cohort] who delivered a non-premature baby with a birth weight > 2,500 g in Munich metropolitan area were included. We assessed PM_2.5_, PM_2.5_ absorbance (which depends on the blackness of PM_2.5_, a marker of traffic-related air pollution), and nitrogen dioxide levels using a land-use regression model, taking into account the type and length of roads, population density, land coverage around the home address, and temporal variations in pollution during pregnancy. Using Poisson regression, we estimated prevalence ratios (PR) of birth weight < 3,000 g, adjusted for gestational duration, sex, maternal smoking, height, weight, and education.

**Results:**

Exposure was defined for 1,016 births. Taking the lowest quartile of exposure during pregnancy as a reference, the PR of birth weight < 3,000 g associated with the highest quartile was 1.7 for PM_2.5_ [95% confidence interval (CI), 1.2–2.7], 1.8 for PM_2.5_ absorbance (95% CI, 1.1–2.7), and 1.2 for NO_2_ (95% CI, 0.7–1.7). The PR associated with an increase of 1 μg/m^3^ in PM_2.5_ levels was 1.13 (95% CI, 1.00–1.29).

**Conclusion:**

Increases in PM_2.5_ levels and PM_2.5_ absorbance were associated with decreases in term birth weight. Traffic-related air pollutants may have adverse effects on birth weight.

Particulate matter (PM) is a major family of atmospheric pollutants (National Center for Environmental Assessment 2004). Fine PM (PM with an aerodynamic diameter < 2.5 μm; PM_2.5_) and, perhaps to a greater extent, ultra-fine particles (PM < 0.1 μm) can penetrate the innermost region of the lungs, and a fraction of them can cross the lung epithelium and enter the blood circulation (Kreyling et al. 2002). Several epidemiologic studies have reported associations between PM levels—most often total suspended particles (TSP) and PM < 10 μm in aerodynamic diameter (PM_10_)—around the maternal home address during pregnancy with offspring birth weight (reviewed by Glinianaia et al. 2004; Šrám et al. 2005). Few studies assessed exposure to PM_2.5_ (Basu et al. 2004; Bell et al. 2007; Dejmek et al. 2000; Jedrychowski et al. 2004; Parker et al. 2005). Four of these studies reported a decrease in term birth weight in relation to maternal exposure to PM_2.5_ during pregnancy; exposure was assessed using individual dosimeters carried 48 hr during pregnancy (Jedrychowski et al. 2004), from the pregnancy-average of the measurements of the air quality monitoring stations within an 8-km radius from the home address (Basu et al. 2004; Parker et al. 2005), or of all the measurement stations located in the county of residence of the woman (Bell et al. 2007).

Fine particles are composed of nonorganic compounds (sulfate, nitrate, ammonium and hydrogen ions, certain transition metals), elemental carbon, organic species including poly-cyclic aromatic hydrocarbons (PAHs) and many other families (National Center for Environmental Assessment 2004; Schauer et al. 1999, 2002). Vehicular traffic is one of the major sources of fine particles. Nitrogen dioxide, PM_2.5_ mass concentration, and also PM_2.5_ absorbance are possible markers of traffic-related pollution (Janssen et al. 2001). More specifically, PM_2.5_ absorbance is a measure of the blackness of PM_2.5_, which strongly depends on the presence of elemental carbon in PM_2.5_ (Janssen et al. 2001; Kinney et al. 2000). Because elemental carbon represents a major fraction of diesel motor exhausts (Lloyd and Cackette 2001; Schauer et al. 1999), PM_2.5_ absorbance is considered a sensitive marker of air pollution due to diesel engines and truck traffic (Janssen et al. 2001; Kinney et al. 2000) and is probably a more sensitive marker of traffic-related pollution than PM_2.5_ (Cyrys et al. 2003; Kinney et al. 2000; Roemer and van Wijnen 2001). Diesel exhaust (Lloyd and Cackette 2001) has been shown in experimental animal studies to be a possible mutagenic agent, to cause allergic and nonallergic respiratory diseases (Krzyzanowski et al. 2005; Pope and Dockery 2006), to be a possible reprotoxicant, and to act as an endocrine disruptor (Takeda et al. 2004; Tsukue et al. 2002; Yoshida et al. 2006). No epidemiologic study has described the association between PM absorbance and birth weight.

With a few exceptions (Choi et al. 2006; Jedrychowski et al. 2004; Wilhelm and Ritz 2003), most epidemiologic studies on the influence of PM or traffic-related pollutants on intrauterine growth restriction relied on birth weight certificates for the collection of birth weight and adjustment factors, whereas exposure was assessed from the background monitoring stations closest to the home address of the mother at the time of delivery. This design has several limitations: Factors known to strongly influence birth weight—such as maternal smoking, weight, or height, not always or accurately available in birth certificates—could not always be controlled for, not allowing researchers to discard confounding (Glinianaia et al. 2004). Exposure misclassification is also a concern: First, pregnancy is often a time to change address, so the exposure levels around the home address at the time of birth might not match exposure levels around the home address during pregnancy for a number of women. Second, all women living within a distance of up to several kilometers around a monitoring station are assumed to be exposed to the pollutants’ levels measured by the station. To limit exposure misclassification, one may prefer to exclude women living far away from monitoring stations (Wilhelm and Ritz 2005); however, monitoring stations are often located at places where population density is higher, and hence air pollution levels are higher. Therefore, if unmeasured environmental or social factors influencing birth weight also varied with distance from monitoring stations, selection bias might occur in studies restricted to subjects living close to monitoring stations. This dilemma between exposure mis-classification and possible selection bias could be avoided by using alternative approaches to model exposure, such as land-use regression or dispersion modeling, which allow modeling of fine spatial contrasts in pollution levels in an area considered as a whole, using information on sources of pollution (Nieuwenhuijsen et al. 2006).

Within a cohort conducted in the Munich metropolitan area (Bavaria), we aimed to characterize the influence of maternal exposure to PM_2.5_, PM_2.5_ absorbance, and NO_2_ during pregnancy on the birth weight of offspring at term, using a land-use regression exposure model and taking into account factors known to influence intrauterine growth.

## Methods

### Population

In the Munich LISA (Influences of Lifestyle Related Factors on the Human Immune System and Development of Allergies in Children) birth cohort, women were included after delivery in six obstetric clinics between January 1998 and January 1999. Exclusion criteria for the mother were, among others, immune-related diseases (including diabetes) and long-term use of medication. Exclusion criteria for the child were birth weight < 2,500 g, gestational duration < 37 completed weeks, congenital malformation, symptomatic neonatal infection, antibiotic medication, and hospitalization or intensive medical care during neonatal period (Gehring et al. 2002). These exclusion criteria had been chosen because the original focus of the cohort was the development of parameters of the immune system, which might be associated with prematurity or low birth weight. We excluded twin births and women who changed home during pregnancy because we did not know their previous home address, and hence could not define their exposure.

Gestational duration and birth weight were collected from the child’s health records filled in at birth by the clinic’s midwife. Information on behavioral, health, and sociodemographic factors was collected during an interview with the mother after birth.

The study was approved by the ethics commission of the Landesaerztekammer Bavaria and was carried out in accordance with the international guidelines for the protection of human subjects. Parents or guardians of all subjects gave written informed consent.

### Exposure model

The exposure model was a stochastic (land-use regression) model with a temporal component. It builds on the previously described TRAPCA (Traffic-Related Air Pollution and Childhood Asthma) II model (Morgenstern et al. 2007). This model is itself an extension and adaptation to part of the Munich metropolitan area of a model previously developed for Munich to study the relation between air pollutants and chronic respiratory diseases in childhood (Brauer et al. 2003; Gehring et al. 2002).

#### Spatial component

**Figure 1:**
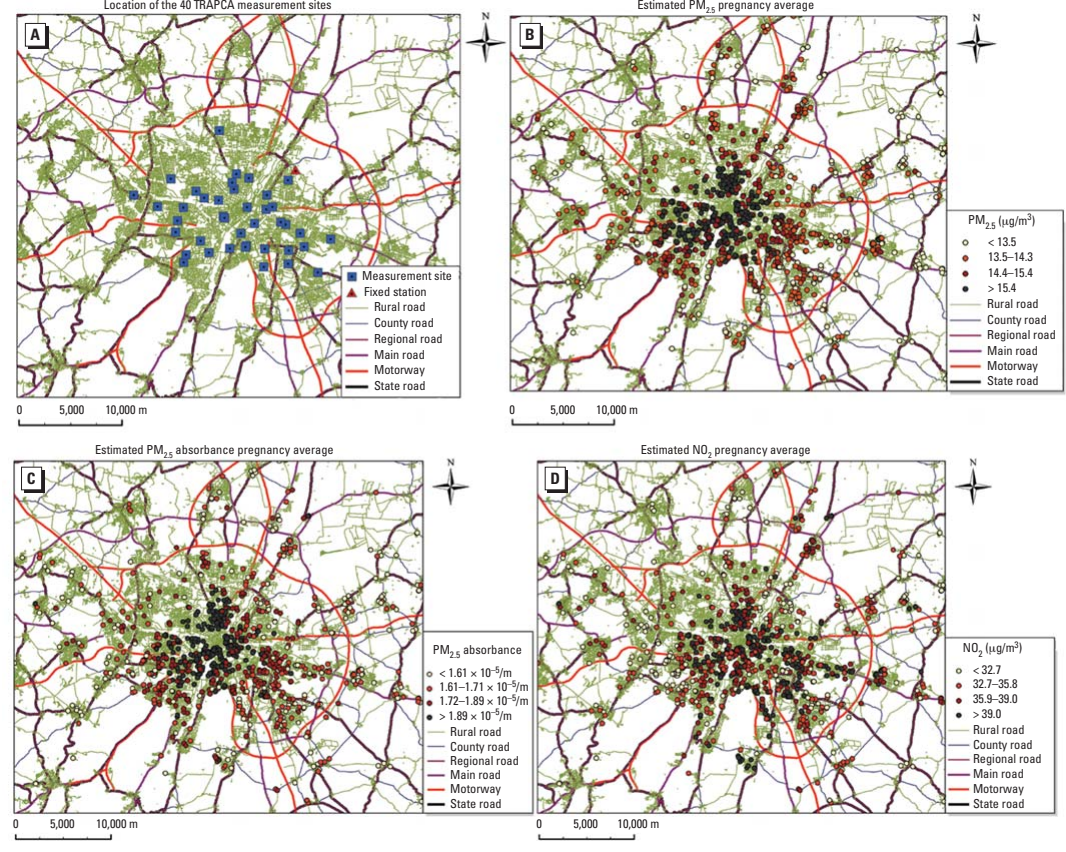
Map of the study area indicating (*A*) the location of the 40 TRAPCA measurement sites and of Johanneskirchen fixed air quality monitoring station (used to seasonalize the exposure model), and (*B,C,D*) the home addresses of the women during pregnancy and the estimated exposure levels (pregnancy averages). (*B*) PM_2.5_ levels; (*C*) PM_2.5_ absorbance; (*D*) NO_2_ levels. To improve readability, the study area furthest from the city center was not represented.

The TRAPCA II model (Morgenstern et al. 2007) was built using four 2-week measurement campaigns at 40 background or traffic sites located in the city of Munich (Figure 1A). The measurements were conducted between March 1999 and July 2000. PM_2.5_ concentration was measured using Harvard impactors (Air Diagnostics and Engineering Inc., Naples, ME, USA); PM_2.5_ absorbance was assessed from the reflectance of the particulate filters by M43D Smoke Stain Reflectometer (Diffusion Systems Ltd., Hanwell, UK) (Hoek et al. 2002), and NO_2_ concentrations by Palmes tubes (Cyrys et al. 2000). A quality control procedure for PM_2.5_ and PM_2.5_ absorbance was conducted (Hoek et al. 2002). For each pollutant, a linear model was fitted with a subset of the following geographic characteristics as covariates: distance of measurement site to each type of road, length of each type of road within various buffers around the site, land coverage, population and household density (within a given ZIP code). The model precision was estimated by cross-validation (Morgenstern et al. 2007). The values of the geographic characteristics corresponding to each home address of a woman in the cohort were retrieved using a geographic information system (ArcGIS 9.1; ESRI, Redlands, CA) and the linear models (consisting of a set of covariates and the corresponding parameters’ values) defined from the 40 measurement sites were applied to the home addresses.

#### Temporal component

These spatial exposure estimates are yearly averages that do not allow testing for a higher susceptibility to atmospheric pollutants during a given trimester of pregnancy. To seasonalize our exposure model (i.e., include a temporal component depending on the conception and delivery dates), we applied the temporal variations observed in one background station in Munich operated by the Bavarian Environmental Protection Agency to the exposure estimate. Of the two background stations operating during the study period, one is located 60 m away from a busy road, and one is in a location distant from an important source of traffic in the suburbs of Munich (Johanneskirchen station; Figure 1A), which is the one we used to build the temporal component of our model. For NO_2_, this was done by averaging the NO_2_ daily mean levels over the pregnancy of each woman, by dividing this average by the average NO_2_ level during the TRAPCA measurement campaign from 1999–2000, and multiplying the corresponding coefficient by the NO_2_ estimate from the TRAPCA II spatial model. PM_2.5_ levels were not measured in Munich during the period corresponding to the pregnancies of the included mothers. To seasonalize the PM_2.5_ estimate, we supposed that temporal variations in PM_2.5_ were similar to that in larger PM measured in the background monitoring station. PM_10_ values were available only from February 2000 onward; values before this date were estimated from the TSP concentration, assuming a conversion factor of 1/1.2 = 0.833 from TSP to PM_10_ (Council of the European Union 1999). For PM_2.5_ absorbance, we assumed that temporal variations were parallel to temporal variations in NO_2_. Using the same approach, we also estimated trimester-specific exposure variables.

### Relation between exposure and birth weight

#### Poisson model

All statistical analyses were conducted using Stata 9.2 statistical package (StataCorp., College Station, TX, USA). Term birth weight was dichotomized using an arbitrary cut-off at 3,000 g. In addition, our *a priori* choice was to analyze birth weight as a continuous outcome, which did not turn out to be associated with air pollutants (data not shown). The exclusion of birth weights < 2,500 g and the relatively low sample size let us *a priori* discard low birth weight and small for gestational age as relevant health outcomes. Given the relatively high frequency of birth weights < 3,000 g, we chose to avoid estimating odds ratios and estimated prevalence ratios (PR). Because log-binomial models failed to converge, we used a Poisson model (Greenland 2004; Spiegelman and Hertzmark 2005) with a maximum likelihood estimator. Confidence intervals (CIs) were constructed by bootstrap.

Adjustment factors were chosen from *a priori* knowledge and hypotheses. However, to limit the number of parameters to estimate, we did not retain maternal passive smoking and age as adjustment factors, which modified the estimated PR associated with pollutants by < 5%. The coding of continuous factors was defined using non- and semiparametric modeling (Slama and Werwatz 2005).

To identify possible windows of susceptibility during pregnancy or before its start, we fitted models with trimester-specific exposure variables. Because the trimester-specific exposure variables relative to a given pollutant were correlated, we also fitted models adjusted for all trimester-specific variables simultaneously.

#### Sensitivity analysis

To quantify possible selection bias due to the noninclusion of children with a birth weight < 2,500 g, we performed a sensitivity analysis using a bootstrap approach (Efron and Tibshirani 1993; Lash and Fink 2003). We expected about 2% of nonpremature children with a birth weight < 2,500 g (Charles MA, Slama R, personal communication), which would correspond to about 20 extra children in our study of about 1,000 births. At each replication, we drew at random with replacement 20 children among those with a birth weight between 2,500 and 2,750 g, and merged these 20 observations with the original data set including 987 observations with a birth weight > 2,500 g and with no missing data on covariates. We then constituted each bootstrap sample by drawing at random with replacement 1,007 observations from this data set of 1,007 observations. This approach therefore assumed that children with a birth weight < 2,500 g would have been similar to those with a birth weight between 2,500 and 2,750 g. The Poisson models were estimated from each bootstrap sample. The bootstrap PR corresponded to the median PR observed among 1,000 replications, and the 95% CI to the empirical 2.5th and 97.5th percentiles of the distribution of the PR.

## Results

### Study population

Among the 1,467 nonpremature newborns from the Munich LISA cohort, 1,287 lived in the Munich metropolitan area, and 1,284 birth addresses were successfully geocoded. We excluded the 27 multiple births; among the remaining 1,257 nonpremature singleton live births that occurred in the study area, we excluded 241 births (19%) corresponding to women who had moved out during pregnancy (*n* = 208) or for whom information on moving was missing (*n* = 33). Mean birth weight of included births was 3,440 g (5th, 50th, and 95th percentiles: 2,800, 3,410 and 4,160 g); 142 children had a birth weight < 3,000 g (14.0%). This proportion was 14.9% in the excluded group of 241 births (percentage comparison test, *p* = 0.7).The characteristics of the study population are given in Table 1.

### Exposure levels

The mean estimated exposure levels averaged over the whole pregnancy (Figure 1B–D) were 14.4 μg/m^3^ for PM_2.5_ (5th, 50th, and 95th percentiles: 11.8, 14.4, and 16.5 μg/m^3^), 1.76 × 10^−5^/m for PM_2.5_ absorbance (5th, 50th, and 95th percentiles: 1.46, 1.72, and 2.14 × 10^−5^/m) and 35.8 μg/m^3^ for NO_2_ (5th, 50th, and 95th percentiles: 28.3, 35.8, and 42.5 μg/m^3^). The correlation between the estimated pollutants’ levels is given Table 2.

There was no evidence of a difference in exposure levels at the home address at the time of delivery between the included population and the 241 excluded births (*p*-value of Student’s *t*-test of comparison of means > 0.5 for all three pollutants).

### Whole pregnancy exposure and term birth weight

The prevalence of birth weights < 3,000 g was 11.4% in the lowest quartile of entire pregnancy exposure to PM_2.5_ and 16.5% in the highest quartile (PR = 1.45; Table 3). After adjustment for the potential confounders, the relative increase in prevalence in the highest quartile was 73% (95% CI, 15 to 169%; Table 3) compared with the lowest quartile of exposure for PM_2.5_, 78% (95% CI, 10 to 170%) for PM_2.5_ absorbance, and 16% (95% CI, –29 to 71%) for NO_2_. The prevalence of birth weights < 3,000 g increased on average by 13% for each increment by 1 μg/m^3^ in PM_2.5_ (95% CI, 0 to 29%), compared with an increment by 6% without adjustment (Table 3). The prevalence of birth weights < 3,000 g increased by 45% for each increment by 0.5 × 10^−5^/m in PM_2.5_ absorbance (95% CI, 6 to 87%), and by 21% for each increment by 10 μg/m^3^ in NO_2_ levels (95% CI, –14 to 68%). There was no evidence of differences in the effect measure of either PM_2.5_ concentration or PM_2.5_ absorbance between male and female newborns (not detailed).

When the pollutants levels were averaged over the 9 months after birth, the estimated increments in the prevalence of birth weight < 3,000 g were lower: 7% for an increase of 1 μg/m^3^ in PM_2.5_, (95% CI, –7 to 22%), 18% for an increase of 0.5 × 10^−5^/m in PM_2.5_ absorbance (95% CI, –16 to 57%), and –2% for an increase of 10 μg/m^3^ in NO_2_ (95% CI, –36 to 38%).

The sensitivity analysis used to study the possible bias due to the exclusion of birth weights < 2,500 g yielded a PR of birth weight < 3,000 g of 1.6 for the highest quartile of PM_2.5_ levels and 1.6 for the highest quartile of PM_2.5_ absorbance (Table 4).

### Disentangling the effects of the three pollutants

**Figure 2:**
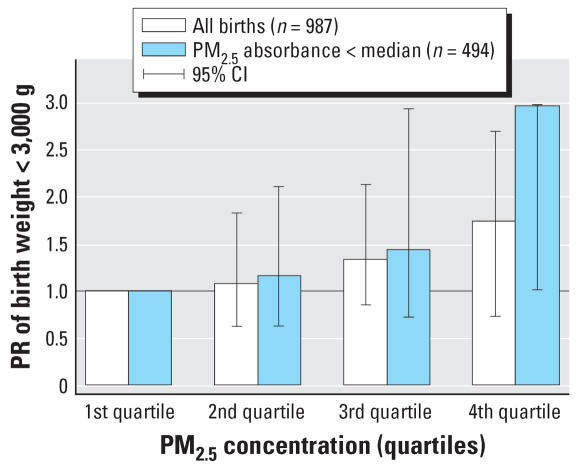
Adjusted PRs of birth weight < 3,000 g associated with PM_2.5_ pregnancy average, in the whole population and in the subgroup in which PM_2.5_ absorbance is below the median value.

When all three pollutants were simultaneously adjusted for, the PR associated with NO_2_ decreased < 1, whereas those associated with PM_2.5_ and PM_2.5_ absorbance exhibited a relative decrease by about 30–50% (Table 3). In single-pollutant models, when we restricted the analyses to observations with a PM_2.5_ absorbance level below the median, the PR associated with PM_2.5_ were close to those observed in the whole population, with broader 95% CI (Figure 2); the PR corresponding to an increase of 1 μg/m^3^ in PM_2.5_ was 1.15 (95% CI, 0.89 to 1.52). The situation was similar for PM_2.5_ absorbance when we restricted the analysis to observations with a PM_2.5_ level below the median (data not shown; PR = 1.67 for an increase of 0.5 × 10^−5^/m in PM_2.5_ absorbance; 95% CI, 0.66 to 3.73).

### Time windows of sensitivity

PM_2.5_ levels during the first and third trimesters of pregnancy were associated with birth weight; when the three trimester-specific exposure variables were simultaneously adjusted for, only the third-trimester PM_2.5_ levels remained associated with birth weight (Table 5). Third-trimester PM_2.5_ levels were highly correlated with the whole pregnancy average (Table 2). In a model simultaneously adjusted for both variables, the PR of birth weight < 3,000 g associated with an increase of 1 μg/m^3^ in PM_2.5_ whole pregnancy average was close to unity (PR = 0.96; 95% CI, 0.75 to 1.19), and that associated with PM_2.5_ third-trimester average varied little (PR = 1.17; 95% CI, 0.98 to 1.40). For PM_2.5_ absorbance, second-trimester exposure was most strongly associated with birth weight (Table 5); the PR associated with second-trimester PM_2.5_ absorbance decreased after adjustment for PM_2.5_ absorbance whole pregnancy average (PR = 1.47; 95% CI, 0.68 to 3.01 for the highest quartile).

The PR corresponding to the exposures during the trimester before pregnancy was close to unity for the three pollutants (not shown).

## Discussion

Among a birth cohort of 1,016 nonpremature children from Bavaria, PM_2.5_ mass concentration and PM_2.5_ absorbance levels around the maternal home address averaged during pregnancy were associated with an increased risk of birth weight < 3,000 g. Our estimates had large uncertainties, as indicated by the CIs. The PRs of birth weight < 3,000 g associated with PM_2.5_ pregnancy-averaged levels decreased after adjustment for PM_2.5_ absorbance, which might be attributed to either PM_2.5_ absorbance’s explaining a part of the estimated effect of PM_2.5_ in single-pollutant models, or to a less efficient estimation of the respective effects of PM_2.5_ and PM_2.5_ absorbance in multipollutant models due to the correlation between both exposure variables. In addition, the PRs of birth weight < 3,000 g associated with PM_2.5_ were similar in the whole population and in the subgroup of subjects with a PM_2.5_ absorbance level below the median, in which confounding by PM_2.5_ absorbance is less likely (Figure 2). Although the CIs were much wider in this subgroup, this gives some evidence that the association between estimated PM_2.5_ levels and birth weight is (at least partly) independent from the association between estimated PM_2.5_ absorbance and birth weight.

### Comparison with former studies

A study in two Czech districts highlighted no association between PM_2.5_ levels averaged over the whole pregnancy and intrauterine growth restriction. In the most polluted district of Teplice, PM_2.5_ levels during the first gestational month were associated with intrauterine growth restriction (Dejmek et al. 1999, 2000). The fact that PM_2.5_ levels were assessed at one monitoring station in each district implied that the exposure model captured only temporal but not spatial variations in air pollution. In a cohort study in Poland among 362 nonsmoking women (median personal exposure, 36 μg/m^3^), an association between personal PM_2.5_ levels and birth weight adjusted for gestational duration and passive smoking assessed by questionnaire has been reported (Jedrychowski et al. 2004). Exposure had been assessed using active air samplers carried by the woman for two consecutive days during the second trimester of pregnancy. From linear regression models in which exposure was log-transformed, Jedrychowski et al. (2004) estimated a decrease by 140 g in mean birth weight when exposure increased from a level of 10 μg/m^3^ up to a level of 50 μg/m^3^, which on average corresponds to a decrease by 3.5 g for each increase of 1 μg/m^3^ in PM_2.5_ exposure. A study in California among 18,247 children born at 40 weeks’ gestation by mothers living < 8 km away from an air monitoring station (Parker et al. 2005) reported an adjusted decrease by 3.8 g in mean birth weight with each increase of 1 μg/m^3^ in PM_2.5_ pregnancy average (95% CI, 2.2 to 5.5 g). In a study in Connecticut and Massachusetts (USA), an increase of 1 μg/m^3^ in PM_2.5_ pregnancy average was associated with an adjusted decrease of 6.7 g (95% CI, 5.6 to 7.8 g) in mean birth weight (Bell et al. 2007). If we assume that the effect of maternal smoking during pregnancy corresponds to a decrease of 10–15 g in birth weight by cigarette smoked each day, in these former studies, the effect of smoking one cigarette per day corresponded to the estimated effect of an increase in PM_2.5_ concentration of 1.5 (Bell et al. 2007) to 4 μg/m^3^ (Parker et al. 2005). In our study, 4% of the pregnant women smoked > 5 cigarettes/day, and an increase of 10 cigarettes/day in maternal smoking was associated with an increase of 66% in the prevalence of birth weight < 3,000 g (95% CI, 5 to 120%), so that the estimated effect of an increase of 1 μg/m^3^ in PM_2.5_ corresponded to that of smoking two to three cigarettes per day. Therefore, the estimated amplitude of the association between PM_2.5_ and birth weight relative to the effect of smoking appears bigger in our study than in the former studies; this comparison is, however, limited by the wide CI of our estimates and by the different exposure assessment methodologies, exposure levels, and pollution mix in the compared studies.

### Time windows of sensitivity

In the California study, there was no evidence for the trimester-specific effect estimates of PM_2.5_ (not adjusted for the other trimesters’ levels) to be clearly stronger for one specific trimester (Parker et al. 2005). In the study in Connecticut and Massachusetts, low birth weight was associated with second- and third-trimester PM_2.5_ levels (Bell et al. 2007). In our study, the strongest associations with birth weight were estimated for the first- and third-trimester PM_2.5_ levels. Our model simultaneously adjusted for all trimester-specific exposure variables tended to suggest that a part of the apparent effect of first-trimester exposure was indeed caused by third-trimester exposure. However, we urge caution in interpreting these results as clear evidence of the existence of a specific window of sensitivity to PM_2.5_ during pregnancy. Indeed, third-trimester PM_2.5_ averages happened to be more strongly correlated with PM_2.5_ pregnancy averages than were first- and second-trimester PM_2.5_ averages (Table 2; a correlation pattern driven mainly by the temporal variations in air pollution during the study period). Therefore, the stronger association between PM_2.5_ third-trimester averages and birth weight (Table 3) than between first- or second-trimester averages and birth weight would also be expected if the whole pregnancy PM_2.5_ average were the most relevant exposure metric. Similarly, PM_2.5_ absorbance second-trimester average was the trimester-specific variable most strongly correlated to PM_2.5_ absorbance whole pregnancy average (Table 2) and also the most strongly associated with birth weight (Table 3). This should not be seen as strong evidence that PM_2.5_ absorbance second-trimester levels are more detrimental to birth weight than the whole pregnancy average—all the more because the model including both exposure variables did not highlight a clearly stronger association with birth weight for one variable or the other. The temporal pattern of the association between trimester-specific NO_2_ averages and birth weight was similar to that of PM_2.5_ absorbance, which was expected because both pollutants shared the same temporal component in our exposure model.

Our results were not in favor of a strong association between preconceptional air pollution levels and birth weight.

### Study population

We excluded about 19% of the cohort members living in the Munich metropolitan area, corresponding to subjects who were likely to have changed home address during pregnancy, because we had no information on their previous addresses. These subjects did not differ from those included in terms of birth weight nor exposure levels at the home address at birth, so this exclusion is unlikely to have entailed a selection bias. Birth weights < 2,500 g may represent about 2% of term births and 10% of infants with birth weight < 3,000 g (Charles MA, Slama R, personal communication); these were not included in the LISA cohort. Our sensitivity analysis tended to indicate that had birth weights < 2,500 g been included, the associations between air pollutant levels and birth weight may have been somewhat weaker, without substantial alteration of the monotonous association. This tends to discard the exclusion of birth weights < 2,500 g as a major source of bias.

### Confounding

We controlled for several factors influencing birth weight, including maternal smoking, height, weight, and maternal education. Women with diabetes were excluded, and we checked that passive smoking assessed by questionnaire, maternal age, and income entailed no confounding. Adjustment did have an impact on the estimated effect of air pollutants coded as continuous factors: The relative increase in the prevalence of birth weight < 3,000 g associated with PM_2.5_ doubled after adjustment; it increased by 80% for PM_2.5_ absorbance. This increase was not attributed to the exclusion of observations with missing values on an adjustment variable (not shown). The factors that, when removed from the adjusted model, entailed the strongest decrease in the PR of birth weight < 3,000 g associated with PM_2.5_ were maternal height, education, and gestational duration. The fact that the pollutants’ levels averaged over the 9 months after birth tended to be less strongly associated with birth weight than the pregnancy averages could be seen as a further argument that residual confounding is unlikely.

Season of conception was strongly associated with air pollution levels, with pregnancy-averaged PM_2.5_ levels being highest for newborns conceived between April and September, and pregnancy-averaged PM_2.5_ absorbance being highest for conceptions occurring between July and December. However, we did not treat season as a confounder. We believed that, apart from chlorination by-products in drinking water (Lewis et al. 2006), there is currently little evidence for factors other than atmospheric pollutants varying with season influencing birth weight; this contrasts with studies on air pollution and mortality, in which season can be seen as a confounder because of its effect on mortality partly mediated by factors with strong seasonal variations such as temperature or occurrence of influenza epidemics. Moreover, the strong correlation between season and exposure, particularly for the trimester-specific exposure variables, was likely to make the estimates of our regression models adjusted for season instable. In our study, adjustment for season had little influence on the estimated effect of pregnancy-averaged exposure: After further adjustment for season of conception, the PRs of birth weight < 3,000 g were 1.68 for the highest quartile of exposure to PM_2.5_ (95% CI, 1.05 to 2.75) and 1.12 for an increase of 1 μg/m^3^ in PM_2.5_ (95% CI, 0.97 to 1.28); for PM_2.5_ absorbance, the corresponding PRs were 1.72 for the highest exposure quartile (95% CI, 1.08 to 2.73) and 1.38 for an increase of 0.5 × 10–5/m (95% CI, 0.96 to 1.86). Adjustment for season had a greater influence on the trimester-specific estimates: After adjustment for season, the PR of birth weight < 3,000 g associated with an increase of 1 μg/m^3^ in third-trimester PM_2.5_ levels simultaneously adjusted for other trimester-specific variables increased to 1.25 (95% CI, 1.04 to 1.50). The mutually adjusted PR associated with an increase of 0.5 × 10–5/m in PM_2.5_ absorbance were 0.93 for first-trimester levels (95% CI, 0.41 to 1.32), 1.14 for second-trimester levels (95% CI, 0.70 to 1.64) and 1.29 for third-trimester levels (95% CI, 0.90 to 1.75) and the PR associated with the highest quartile of PM_2.5_ absorbance were 0.73 (95% CI, 0.38 to 1.38), 2.45 (95% CI, 1.22 to 4.77), and 1.19 (95% CI, 0.60 to 2.48) for the first-, second-, and third-trimester levels, respectively.

### Assessment of exposure to atmospheric pollutants

#### Temporal component

To study exposure windows ranging from 3 to 9 months, we added a temporal component to the exposure model. In doing so, we made several assumptions. First, we assumed that temporal variations in the considered atmospheric pollutants were similar across the metropolitan area. For NO_2_, the pairwise correlations between the daily measurements of the seven background and traffic stations of the local air quality monitoring network ranged from 0.52 to 0.90 (median, 0.75). For PM_2.5_, Gomišček et al. (2004) reported a correlation of 0.79 inPM_2.5_ daily concentrations between a rural and an urban site in Vienna over a 1-year period. Because there was no monitoring of PM_2.5_ and PM_2.5_ absorbance in Munich when the pregnancies took place, we had to assume that temporal variations in PM_2.5_ paralleled variations in PM_10_ (Gehrig and Buchmann 2003) and that total suspended particles were strongly correlated to PM_10_ (Monn et al. 1995). Finally, we assumed that temporal variations in PM_2.5_ absorbance paralleled variations in NO_2_. This assumption is supported by a coefficient of correlation of 0.83 between daily PM_2.5_ absorbance and NO_2_ levels measured at one monitoring station in Erfurt, Germany, from 2001 to 2002 (Cyrys J, personal communication). Although reasonable, these assumptions are likely to have induced exposure misclassification, which we believe to be minor compared with that which would exist had temporal variations in air pollution been ignored. The original exposure estimates (Morgenstern et al. 2007) were strongly correlated with our seasonalized exposure estimates (coefficient of correlation, 0.95 for PM_2.5_, 0.89 for PM_2.5_ absorbance); their associations with birth weight were weaker than with our seasonalized model. For example, the PR of birth weight < 3,000 g associated with an increase of 1 μg/m^3^ in PM_2.5_ levels averaged during pregnancy was 1.10 (95% CI, 0.94 to 1.27) with the nonseasonalized exposure model (Morgenstern et al. 2007), compared with 1.13 with the seasonalized model. For an increase of 0.5 × 10^−5^/m in PM_2.5_ absorbance, the PR was 1.31 with the nonseasonalized model (95% CI, 0.91 to 1.80). This may be seen as empirical evidence of the importance of including temporal trends in land-use regression exposure models when short term effects of exposure are expected.

#### Spatial component

Limitations of our exposure model are that exposures at the work address and during transport were not taken into account. Moreover, the model made the assumption that outdoor pollutant levels were good approximations of personal exposure. This is the case for NO_2_ for homes without indoor combustion sources (Cyrys et al. 2000). Concerning PM_2.5_, a longitudinal exposure assessment study in Amsterdam, the Netherlands, and Helsinki, Finland, reported median coefficients of correlation between individual exposure and outdoor levels assessed in the vicinity of the home ranging from 0.7 to 0.8 for PM_2.5_. Higher correlations were observed for the contents in sulfur element and in sulfate ion of PM_2.5_. For PM_2.5_ absorbance, coefficients of correlation between individual exposure and outdoor levels of 0.8–0.9 have been reported (Brunekreef et al. 2005). Therefore, in this population, outdoor levels in the vicinity of the home were good markers of individual exposure for PM_2.5_ absorbance levels, and probably also for PM_2.5_ of outdoor origin. PM_2.5_ of indoor origin has a different composition and hence possibly different health effects, and thus warrants separate consideration.

Several facts point toward road traffic as a major source of the pollutants that we assessed: first, the association between PM_2.5_ absorbance, a sensible marker of traffic-related air pollution (Janssen et al. 2001; Kinney et al. 2000; Roemer and van Wijnen 2001) and birth weight; second, the fact that length of roads in the vicinity of the home address were predictors of the exposure levels (Morgenstern et al. 2007); third, that road traffic accounts for about 60% of PM_10_ emissions in Munich (Regierung von Oberbayern 2004), a proportion that is probably higher for PM_2.5_ emissions. Possible harmful effects of air pollution due to road traffic on birth weight are further supported by another study (Wilhelm and Ritz 2003) and by the possible effect of PAH on birth weight (Perera et al. 2004a). The respective contributions of gasoline-powered cars, light-duty diesel-powered vehicles, and heavy-duty vehicles in the PM_2.5_ and PM_2.5_ absorbance levels in the study area cannot easily be distinguished. On a per-vehicle basis, the emission rate of fine PM and elemental carbon increases from light-duty gasoline-powered vehicles to light-duty diesel-powered vehicles, heavy-duty vehicles, and nonroad engines such as bulldozers (Lloyd and Cackette 2001). For instance, an average emission rate of 0.8 mg elemental carbon per kilometer driven has been reported for a group of catalyst-equipped gasoline-powered cars (Schauer et al. 2002), compared with 56 mg/km for medium-duty diesel trucks (Schauer et al. 1999). However, the overall contribution in elemental carbon in fine PM of each type of vehicle depends on the vehicle mix; because most of the German vehicle fleet is composed of gasoline-powered cars, these may also contribute significantly to the estimated PM_2.5_ absorbance levels. In Munich in 2000, 17% of the fleet of 679,000 light-duty vehicles registered in the city was diesel-powered (Munich City Statistical Office, personal communication).

NO_2_ was weakly associated with birth weight, and any association disappeared after control for PM_2.5_ levels, although the wide CIs do not allow us to discard an association between NO_2_ levels and birth weight independently of PM_2.5_ levels. Assuming that the associations observed with PM_2.5_ and PM_2.5_ absorbance reflected causal effects, the fact that NO_2_ was not clearly associated with birth weight could be attributed to our exposure model being less accurate for NO_2_ than for the other pollutants; alternatively, it may also be attributed to NO_2_ being a less sensitive marker of the pollutants influencing birth weight than PM_2.5_ and PM_2.5_ absorbance. Previous work on the TRAPCA model for the city of Munich indicated a somewhat greater proportion of variance explained by traffic variables for PM_2.5_ absorbance (Brauer et al. 2003) than for NO_2_ and PM_2.5_ (Cyrys et al. 2005).

### Statistical modeling

Simulations tend to indicate that bias in the maximum likelihood estimates of the parameters of a logistic regression model and their variance (“overfitting”) may be a concern if there are < 10 events per variable (Harrell 2001; Peduzzi et al. 1996), whereas other researchers consider that bias remains infrequent with as few as 5–9 events per variable in the model (Vittinghoff and McCulloch 2007). In our study, the number of cases in the adjusted models was 139, and the number of adjustment variables exceeded 14 in multipollutant models as well as in models including simultaneously all trimester-specific exposure variables. Thus, if we consider that the conclusions of these simulations (Peduzzi et al. 1996; Vittinghoff and McCulloch 2007) also hold for Poisson regression, one should consider with caution the estimates from our models including all pollutants simultaneously. The use of bootstrap to estimate confidence intervals may have reduced any effect of overfitting.

### Possible biological mechanisms

Several biological mechanisms leading to intrauterine growth restriction have been identified, among which are placental or fetal hypoxia, reduced maternal–placental blood flow, inflammatory processes, genetic (Infante-Rivard et al. 2006) or epigenetic (Miozzo and Simoni 2002) changes, viral infections and endocrine disruption (Kanaka-Gantenbein et al. 2003). Therefore, an effect of air pollutants on the placenta, the embryo, the maternal immunologic system, or the maternal hypothalamic–ovarian axis might induce intrauterine growth restriction. There is some evidence that atmospheric pollutants reach some of the target organs or interfere with the above-mentioned physiologic systems. For example, exposure to PAHs during pregnancy has been shown to alter maternal serum progesterone and estrogen levels, as well as fetal survival in F-344 rats (Archibong et al. 2002), and diesel exhausts are likely to be endocrine disruptors in rodents (Takeda et al. 2004; Tsukue et al. 2002). PM_2.5_ levels might be associated with altered plasma viscosity (Peters et al. 1997), markers of inflammation such as C-reactive protein (Dubowsky et al. 2006), and blood pressure (Brook 2005) among susceptible human populations. All these effects might influence intrauterine growth; however, pregnant women may differ from these populations in terms of immunologic status, heart rate, plasma viscosity, and insulin resistance (Kaaja and Greer 2005), so that it is unclear whether such possible effects are enhanced or inhibited among pregnant women.

Several compounds of the PAH family are present in particles stemming from road traffic (Schauer et al. 1999, 2002). Personal exposure to PAHs (Whyatt et al. 1998) and maternal PAH–DNA adducts (Perera et al. 2004b) have been correlated with the presence of PAH–DNA adducts in umbilical white blood cells. Atmospheric PAH levels have been associated with altered intrauterine growth in some populations (Choi et al. 2006; Dejmek et al. 2000). An association between the presence of PAH–DNA adducts in umbilical white blood cells and birth weight has also been reported in Poland (Perera et al. 1998), but not in Manhattan, New York (Perera et al. 2005).

Tobacco smoke—which contains particles peaking between 0.3 and 0.4 μm in diameter (Kleeman et al. 1999) and PAHs, among many other families of pollutants—influences intrauterine growth restriction and has been shown to be associated with altered umbilical and uterine artery blood flow (Albuquerque et al. 2004) and altered placental structure and function (Zdravkovic et al. 2005).

Overall, there is therefore suggestive evidence that PM_2.5_ and traffic-related air pollutants interfere with several key organs and functions implied in intrauterine growth.

## Conclusions

We highlighted an increased prevalence of birth weights < 3,000 g in association with estimated outdoor PM_2.5_ levels and PM_2.5_ absorbance at the home address of the mother during pregnancy. These associations were monotonous, and unlikely to be attributed to confounding by the main factors known to influence birth weight. This is, to our knowledge, the first study on the influence of PM levels on term birth weight that uses a GIS-based land-use regression model to assess exposure, and the first to show that PM_2.5_ absorbance may be associated with decreases in birth weight. Except for a study among a birth cohort from 1946 (Bobak 2000) and of an ecologic Finnish study (Hansteen et al. 1998), no study had so far been published on populations from Western Europe. Overall, this study indicates that traffic-related air pollutants influence term birth weight.

## Figures and Tables

**Table 1:** Characteristics of the included 1,016 nonpremature singleton births.

		Birth weight	
Characteristic	No. (%)	Mean (g)	Birth weight < 3,000 g (%)
Gestational duration (weeks)			
37	39 (4)	3,040	44
38	89 (9)	3,170	30
39	182 (18)	3,310	19
40	403 (40)	3,460	12
41	210 (21)	3,590	6
≥ 42	93 (9)	3,670	2
Sex of the child			
Female	478 (47)	3,370	18
Male	538 (53)	3,500	10
Period of conception			
January–March	244 (24)	3,440	13
April–June	265 (26)	3,420	13
July–September	246 (24)	3,450	15
October–December	261 (26)	3,440	15
Maternal parity before the index pregnancy			
0	540 (53)	3,380	15
≥ 1	476 (47)	3,500	12
Maternal tobacco smoking during 3rd trimester			
0	923 (91)	3,450	13
1–10 cigarettes/day	75 (7)	3,340	17
> 10 cigarettes/day	14 (1)	3,200	43
Maternal passive smoking during pregnancy			
No	803 (83)	3,440	13
Yes	166 (17)	3,400	19
Maternal education			
Up to 9 years of school attendance	80 (8)	3,420	20
10 years, degree	292 (29)	3,420	17
Vocational school (*Fachschule*)	66 (7)	3,430	11
High school (*Abitur*)	569 (57)	3,450	12
Maternal height (cm)			
≤ 160	99 (10)	3,240	28
161–170	547 (55)	3,420	14
171–180	337 (34)	3,510	11
> 180	18 (2)	3,680	0
Maternal prepregnancy weight (kg)			
≤ 50	59 (6)	3,180	32
51–60	393 (39)	3,400	16
61–70	375 (38)	3,470	11
71–80	105 (10)	3,550	10
> 80	71 (7)	3,550	10
Maternal prepregnancy BMI (kg/m^2^)			
≤ 18	31 (3)	3,360	13
18 < BMI ≤ 20	188 (19)	3,350	20
20 < BMI ≤ 22.5	431 (42)	3,450	14
22.5 < BMI ≤ 25	189 (19)	3,480	11
25 < BMI ≤ 30	117 (12)	3,490	11
30 < BMI	60 (6)	3,460	10

BMI, body mass index.

**Table 2:** Coefficient of correlation between the estimated air pollutants’ levels.

	PM_2.5_				PM_2.5_ absorbance				NO_2_			
	Pregnancy average	1st trimester	2nd trimester	3rd trimester	Pregnancy average	1st trimester	2nd trimester	3rd trimester	Pregnancy average	1st trimester	2nd trimester	3rd trimester
PM_2.5_												
Pregnancy average	1											
1st trimester	0.85	1										
2nd trimester	0.77	0.40	1									
3rd trimester	0.87	0.68	0.51	1								
PM_2.5_ absorbance												
Pregnancy average	0.69	0.68	0.41	0.62	1							
1st trimester	0.33	0.27	0.08	0.48	0.54	1						
2nd trimester	0.48	0.53	0.29	0.36	0.84	0.32	1					
3rd trimester	0.52	0.51	0.41	0.37	0.55	–0.26	0.31	1				
NO_2_												
Pregnancy average	0.45	0.48	0.23	0.39	0.67	0.29	0.61	0.40	1			
1st trimester	0.18	0.15	–0.03*	0.33	0.34	0.84	0.19	–0.34	0.54	1		
2nd trimester	0.32	0.41	0.17	0.21	0.63	0.16	0.85	0.21	0.84	0.33	1	
3rd trimester	0.37	0.39	0.30	0.23	0.36	–0.39	0.17	0.88	0.59	–0.21	0.34	1

* *p* = 0.31. All other *p*-values testing equality to 0 are < 0.01.

**Table 3:** PRs of birth weight < 3,000 g associated with the estimated exposure levels to atmospheric pollutants averaged during the whole pregnancy, among 1,016 children from the LISA cohort born in Munich.

	Single-pollutant models							Multipollutant models^a^		
	Unadjusted models				Adjusted models^b^			Adjusted models^b^		
Air pollutants level	No.	BW < 3,000 g (%)	PR	95% CI^c^	No.	PR	95% CI^c^	No.	PR	95% CI^c^
PM_2.5_ (μg/m^3^)										
1st quartile (7.2–13.5)	254	11.4	1		247	1		247	1	
2nd quartile (13.5–14.4)	254	12.2	1.07	0.65–1.73	242	1.08	0.63–1.82	242	1.01	0.57–1.85
3rd quartile (14.4–15.4)	254	15.8	1.38	0.91–2.09	251	1.34	0.86–2.13	251	1.12	0.64–1.87
4th quartile (15.41–17.5)	254	16.5	1.45	0.92–2.25	247	1.73	1.15–2.69	247	1.36	0.72–2.45
*Continuous coding (increase of 1* μ*g/m^3^)*	1,016	14.0	1.06	0.95–1.19	987	1.13	1.00–1.29	987	1.07	0.91–1.26
PM_2.5_ absorbance (10^−5^/m)										
1st quartile (1.29–1.61)	254	10.6	1		245	1		245	1	
2nd quartile (1.61–1.72)	254	12.6	1.19	0.74–1.99	249	1.21	0.73–1.97	249	1.19	0.70–2.01
3rd quartile (1.72–1.89)	254	16.5	1.56	0.98–2.50	247	1.63	0.98–2.57	247	1.55	0.80–2.80
4th quartile (1.89–3.10)	254	16.1	1.52	0.96–2.46	246	1.78	1.10–2.70	246	1.46	0.67–2.90
*Continuous coding (increase of 0.5* × *10*^−^*^5^/m)*	1,016	14.0	1.25	0.90–1.70	987	1.45	1.06–1.87	987	1.33	0.76–2.38
NO_2_ (μg/m^3^)										
1st quartile (23.6–32.7)	254	13.8	1		247	1		247	1	
2nd quartile (32.7–35.8)	254	11.0	0.80	0.51–1.24	249	0.80	0.52–1.28	249	0.70	0.43–1.24
3rd quartile (35.8–39.0)	254	17.3	1.26	0.86–1.95	246	1.32	0.86–2.09	246	1.04	0.59–1.79
4th quartile (39.0–60.8)	254	13.8	1.00	0.64–1.58	245	1.16	0.71–1.71	245	0.84	0.47–1.45
*Continuous coding (increase of 10* μ*g/m^3^)*	1,016	14.0	1.07	0.77–1.50	987	1.21	0.86–1.68	987	0.95	0.57–1.64

BW, birth weight.

a Two separate models were fitted: one including all three pollutants coded in quartiles (dummy variables), the other including all three pollutants as continuous terms. Both models were adjusted for the covariates noted below.

b PRs were adjusted for gestational duration (continuous variable), sex of the child, maternal smoking (continuous variable), parity (0, ≥ 1 previous birth), maternal education, maternal size (broken stick variables with a threshold at 160 cm), and prepregnancy weight (broken stick variables with a threshold at 60 kg).

c Bootstrap CIs (bias-corrected and accelerated).

**Table 4:** Sensitivity analysis - Bootstrap PRs of birth weight < 3,000 g and empirical 95% CIs associated with the estimated exposure levels to atmospheric pollutants averaged during pregnancy.

Air pollutants levels	No.	Bootstrap PR^a^	Empirical 95% CI^b^
PM_2.5_ (μg/m^3^)			
1st quartile (7.2–13.5)		1	
2nd quartile (13.5–14.4)		0.98	0.63–1.61
3rd quartile (14.4–15.4)		1.22	0.82–2.02
4th quartile (15.41–17.5)		1.57	1.02–2.57
*Continuous coding (increase of 1* μ*g/m^3^)*	1,007	1.11	0.98–1.27
PM_2.5_ absorbance (10^−5^ m)			
1st quartile (1.29–1.61)		1	
2nd quartile (1.61–1.72)		1.19	0.76–1.91
3rd quartile (1.72–1.89)		1.52	0.99–2.34
4th quartile (1.89–3.10)		1.62	1.06–2.55
*Continuous coding (increase of 0.5 × 10*^−^*^5^/m)*	1,007	1.35	1.01–1.83
NO_2_ (μg/m^3^)			
1st quartile (23.6–32.7)		1	
2nd quartile (32.7–35.8)		0.80	0.51–1.22
3rd quartile (35.8–39.0)		1.32	0.85–2.05
4th quartile (39.0–60.8)		1.14	0.77–1.73
*Continuous coding (increase of 10* μ*g/m^3^)*	1,007	1.16	0.85–1.60

To correct for possible selection bias, children with a birth weight between 2,500 and 2,750 g were oversampled.

a PRs were adjusted for the same variables as in Table 3. Each value corresponds to the median observed over 1,000 bootstrap replications.

b 2.5th and 97.5th percentiles of the distribution of PR over 1,000 bootstrap replications.

**Table 5:** Adjusted PRs of birth weight < 3,000 g according to the estimated exposure levels, for trimester-specific exposure windows, among 1,016 singleton children from the LISA cohort.

	1st trimester			2nd trimester		3rd trimester	
Air pollutant level	No.	PR^a^	95% CI^b^	PR^a^	95% CI^b^	PR^a^	95% CI^b^
PM_2.5_							
Each trimester separately							
1st quartile (lowest)	245	1		1		1	
2nd quartile	246	1.14	0.74–1.96	0.83	0.52–1.32	1.30	0.80–2.17
3rd quartile	249	1.28	0.84–2.10	1.08	0.71–1.60	1.44	0.85–2.27
4th quartile	247	1.65	1.02–2.60	0.94	0.61–1.47	1.90	1.20–2.82
*Continuous coding (increase of 1* μ*g/m^3^)*	987	1.10	0.99–1.20	1.01	0.92–1.12	1.14	1.02–1.24
All trimesters together^c^							
1st quartile	245	1		1		1	
2nd quartile	246	0.97	0.60–1.73	0.75	0.46–1.24	1.34	0.79–2.30
3rd quartile	249	0.98	0.57–1.75	0.86	0.56–1.30	1.48	0.86–2.58
4th quartile	247	1.22	0.71–2.18	0.75	0.48–1.23	1.91	1.00–3.20
*Continuous coding (increase of 1* μ*g/m^3^)*	987	1.03	0.90–1.17	0.94	0.84–1.06	1.14	0.99–1.29
PM_2.5_ absorbance							
Each trimester separately							
1st quartile	249	1		1		1	
2nd quartile	243	1.15	0.73–1.80	1.33	0.85–2.22	1.30	0.85–2.09
3rd quartile	248	1.01	0.61–1.53	1.76	1.07–2.91	0.92	0.55–1.50
4th quartile	247	1.04	0.70–1.57	1.83	1.11–2.81	1.50	1.00–2.27
*Continuous coding (increase of 0.5 × 10*^−^*^5^/m)*	987	1.03	0.82–1.28	1.27	1.04–1.54	1.20	0.98–1.44
All trimesters together^c^							
1st quartile	249	1		1		1	
2nd quartile	243	0.90	0.52–1.58	1.30	0.77–2.16	0.99	0.64–1.62
3rd quartile	248	0.82	0.45–1.31	1.63	0.93–2.73	0.71	0.40–1.20
4th quartile	247	0.88	0.53–1.42	1.99	1.12–3.33	1.14	0.68–1.91
*Continuous coding (increase of 0.5 × 10*^−^*^5^/m)*	987	1.02	0.77–1.29	1.21	0.93–1.54	1.15	0.92–1.42
NO_2_							
Each trimester separately							
1st quartile	248	1		1		1	
2nd quartile	248	1.01	0.67–1.57	0.99	0.62–1.54	1.17	0.73–1.95
3rd quartile	248	1.07	0.71–1.60	1.30	0.79–2.00	1.05	0.64–1.75
4th quartile	243	0.86	0.53–1.30	1.35	0.88–2.11	1.42	0.91–2.22
*Continuous coding (increase of 10* μ*g/m^3^)*	987	0.96	0.73–1.20	1.18	0.95–1.44	1.13	0.91–1.35
All trimesters together^c^							
1st quartile	248	1		1		1	
2nd quartile	248	0.87	0.55–1.45	0.99	0.62–1.58	1.08	0.65–1.86
3rd quartile	248	1.00	0.60–1.64	1.25	0.76–2.02	0.90	0.52–1.62
4th quartile	243	0.81	0.45–1.36	1.38	0.80–2.34	1.25	0.72–2.09
*Continuous coding (increase of 10* μ*g/m^3^)*	987	0.92	0.67–1.22	1.19	0.93–1.51	1.06	0.82–1.30

a PR of birth weight < 3,000 g adjusted for the same variables as in Table 3.

b Bootstrap CIs (bias-corrected and accelerated).

c For each pollutant, the models were adjusted simultaneously for the three trimester-specific exposure variables.
